# Stroke Classification in Table Tennis as a Multi-Label Classification Task with Two Labels Per Stroke

**DOI:** 10.3390/s25030834

**Published:** 2025-01-30

**Authors:** Yuta Fujihara, Tomoyasu Shimada, Xiangbo Kong, Ami Tanaka, Hiroki Nishikawa, Hiroyuki Tomiyama

**Affiliations:** 1 Graduate School of Science and Engineering, Ritsumeikan University, Shiga 525-8577, Japan; ri0080xs@ed.ritsumei.ac.jp (T.S.); a-tanaka@fc.ritsumei.ac.jp (A.T.); ht@fc.ritsumei.ac.jp (H.T.); 2 Faculty of Information Engineering, Toyama Prefectural University, Toyama 939-0398, Japan; kong@pu-toyama.ac.jp; 3 Graduate School of Information Science and Technology, Osaka University, Osaka 565-0871, Japan; nishikawa.hiroki@ist.osaka-u.ac.jp

**Keywords:** table tennis, action recognition, multi-labeling

## Abstract

In table tennis, there are various movements involved in hitting a ball, which are called strokes, and these are an important factor in determining the contents of a game. Therefore, research has been conducted to classify these types of strokes using video gameplay data or inertial sensor information. However, the classification of strokes from actual videos of table tennis is more difficult than general action recognition tasks because many strokes display strong similarity. Therefore, this study proposes a multi-label stroke classification method, assigning multiple classes per stroke. Specifically, multi-labeling is performed by assigning two types of labels—namely the player’s posture and the rotation and velocity of the ball—to one stroke. By changing the head of the action recognition model to adopt multiple outputs for stroke classification, the difficulty in each classification task is reduced and the accuracy is improved. As a result, when performing multi-labeling classification with a conventional action recognition model, the accuracy of the validation data was improved by up to 8.6%, and the accuracy of the test data was improved by up to 18.1%. In addition, when two types of input—namely video and 3D joint coordinates—were used, the accuracy of the validation and test data was higher by 17.1 and 5.4% for 3D joint coordinates, respectively, confirming that 3D joint coordinates are effective.

## 1. Introduction

**Figure 1:**
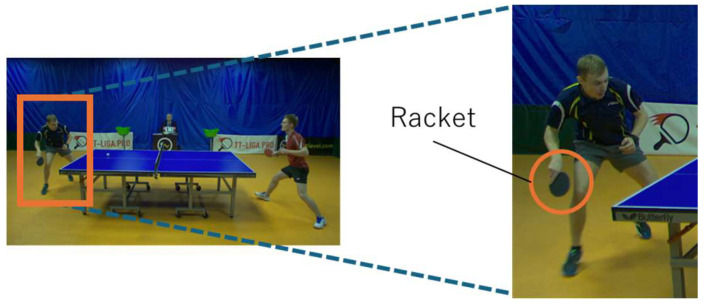
A scene from a table tennis game.

**Figure 2:**
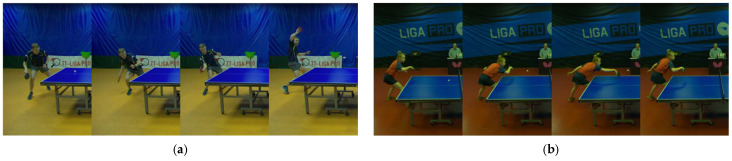
Scenes that the players perform strokes: (**a**) Forehand Topspin, (**b**) Backhand Push.

**Figure A1:**
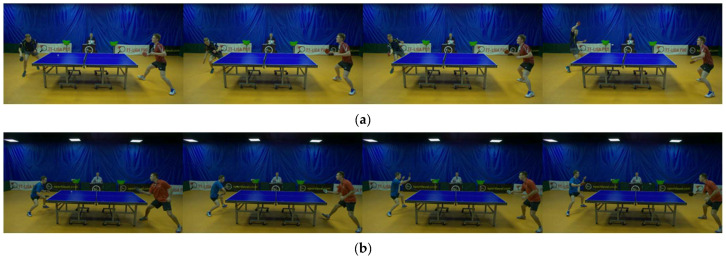

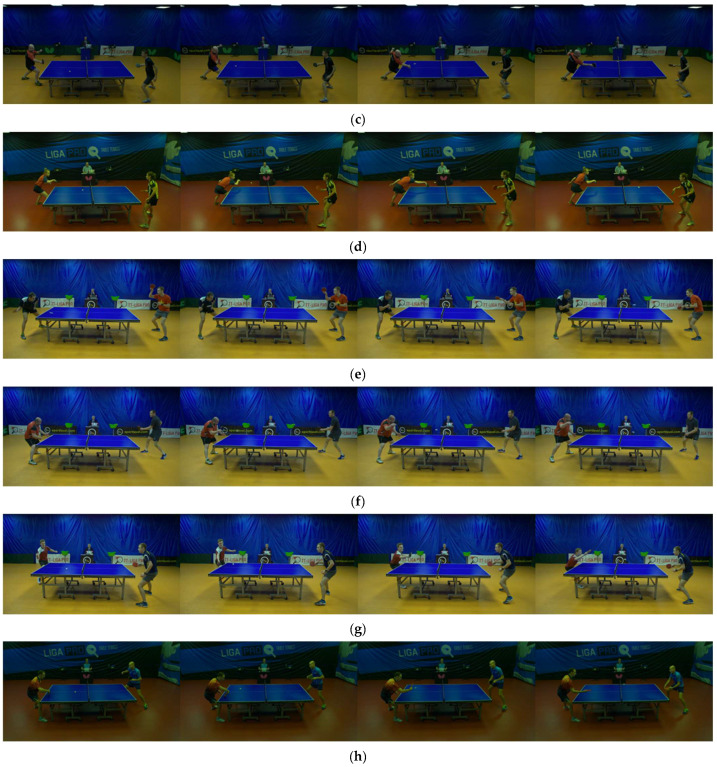
Examples of video used for classification: (**a**) Forehand Topspin, (**b**) Backhand Topspin, (**c**) Forehand Push, (**d**) Backhand Push, (**e**) Forehand Block, (**f**) Backhand Block, (**g**) Forehand Flick, (**h**) Backhand Flick.

In sports, it is highly important to record players’ movements during games to identify areas for improvement and decide on training methods. However, typically, players’ movements during games are recorded and analyzed by humans, and the methods are limited. Therefore, to improve players’ abilities through the more efficient use of their data, sports analysis based on computer vision has been emphasized, such as the collection and analysis of videos and images, and various studies have been conducted. Table tennis is a competitive sport characterized by various hitting techniques called strokes. These strokes vary depending on the ball’s speed and spin. Figure 1 shows a scene from a table tennis game. As shown in this figure, players use rackets to strike the ball across a table divided by a net. The stroke technique is fundamental to gameplay, as players score points through the effective use of different strokes. There are many different types of strokes, but the study [1] defines 10 strokes. Figure 2 shows examples of strokes. Each is a few frames taken from a video of the area around the player performing the stroke. Figure 2a shows a forehand topspin. In a forehand topspin, the player swings the racket up from the bottom and rotates the ball upward with the palm facing the opponent. Figure 2b shows a backhand push. In a backhand push, the racket is pushed forward with the back of the hand facing the racket against the ball on the table. Other strokes are shown in Figure A1 in Appendix A.

Strokes are an important factor in analyzing the contents of a game, and previous research has aimed to classify such strokes through the application of table tennis videos and images [2,3,4]. However, previous studies have not considered videos taken during an actual match; instead, they use videos taken by a camera installed on a table tennis table and acquire data by attaching an inertial sensor to the racket [1]. In recent years, some researchers have proposed stroke classification methods for game videos [5], but such studies are lacking. In this study, we classify strokes using actual game videos in order to establish a stroke classification method that is applicable to such videos. In game videos, players are filmed from various viewpoints, so it is important to extract features that do not depend on the viewpoint. Therefore, we aim to improve the classification performance by extracting the three-dimensional joint coordinates of players from videos and learning them through deep learning.

Table tennis strokes involve the basic motion of swinging one’s arms, but their forms are slightly different depending on how the player spins and how quickly they hit the ball. Nonetheless, most strokes involve largely similar motions, making their classification a difficult task compared to general action recognition tasks [5]. Usually, the behavior of the recognition object is uniquely determined for one input, so it has only one label for one input. Therefore, conventional studies on stroke classification have mainly improved the extraction part of the feature without improving the method of assigning the label or the output. On the other hand, the stroke type is characterized by various independent factors. Specifically, there are two types: One is determined by the surface of the racket where the ball is hit, and the other is determined by the speed and rotation of the hit ball. In this study, we look at the factors that determine strokes and propose a classification method in which we assign two types of labels to each stroke: the player’s posture (forehand/backhand) and the rotation and velocity of the ball. Finally, by combining these classification results, we determine the types of strokes. In this way, stroke classification is considered as a multi-label classification problem, and we seek to reduce the difficulty in the classification task per stroke, thereby improving the classification accuracy. In this method, a general action recognition model is used as the backbone; by branching its head part, the classification results regarding the two outputs are obtained, and these outputs are combined to obtain the final classification result. Since the proposed method improves the accuracy by improving the method of assigning labels, which is completely different from the improvement inside the network, it is expected to further improve the accuracy of the existing action recognition model. The main contributions of this study are as follows.

- Our proposed method of stroke classification improves the accuracy of table tennis analysis;
- We demonstrate that the three-dimensional joint coordinates of players are appropriate inputs for the classification of game videos;
- Several classification methods improve the accuracy via the proposed technique of multi-labeling.

The structure of this paper is as follows. Section 2 describes the related work. Section 3 describes the data used for classification and the multi-label classification method. Section 4 discusses the experiments and results. Finally, Section 5 presents a summary of this paper and future issues.

## 2. Related Work

Various studies have been conducted on stroke classification in table tennis. One approach is to perform classification based on information obtained from inertial sensors. In one study [2], an inertial sensor was attached to the racket to detect and classify strokes based on temporal changes in acceleration. Another study used a smartwatch as a sensor to reduce the load on players during practice due to the widespread use of wearable devices [4]. In [6], the relationship between the type of stroke and the movement of the joint coordinates was experimentally demonstrated. This study reveals which joints affect the velocity and rotation of the ball, and this information can help players to improve their technique. Recently, there have been studies to evaluate recognized strokes. In [7], strokes are scored based on information obtained from sensors, and it is statistically shown that the movements of professional players are more ideal than amateur players. In [8], changes in acceleration during the swing are used to provide theoretical values of what needs to be improved to improve the player’s ability. In [9], serve (the first action to hit the ball at the start of the rally) is evaluated based on information obtained from inertial sensors, and the difference in the movements depending on the type of serve is clarified experimentally. Thus, in recent years, not only the classification of the movements performed in table tennis, but also the research to evaluate the movements and obtain the information that can be fed back to the players has been increasing, and the importance of the classification of the movements as the basis for the evaluation is also increasing.

Another approach is to perform classification based on computer vision. In one study [2], classification was performed by capturing images of players using a camera installed on a table tennis table, followed by using a convolutional neural network to learn the transition of the two-dimensional joint coordinates. In another work [10], a dataset called TTStroke-21, consisting of the strokes of 17 players, was created, and classification was performed on this dataset using videos and optical flows as inputs. In addition, the same group has proposed several classification methods for TTStroke-21 [11,12]. In [11], a network with an attention mechanism added to a three-dimensional convolutional layer was proposed. It was shown that the attention mechanism learned to attract attention to the player who hit the ball in the video, and the accuracy was higher than that demonstrated for a network without an attention mechanism. In [12], an image of the two-dimensional joint coordinates plotted against a black background was used as a new input. The accuracy was improved compared with the input of a raw video only.

Thus, most studies on stroke classification have focused on wearing sensors or collecting data under a specific shooting environment. This is not suitable for the analysis of actual game videos. Several studies have been conducted to address this. In one study [5], a dataset of practice matches and rallies, named “Table Tennis Shots 1.0”, was created, and the strokes in the videos were classified. In another work, a classification method was obtained by replacing the global pooling layer of a three-dimensional CNN with BERT [13] to capture minute temporal variations between strokes. In [14], a dataset was created from videos provided by the International Table Tennis Federation, and classification was performed. In this study, a recognition model focusing on different features was also obtained by preparing a video in which a player was captured and a video in which a player’s arm was captured from a raw video, followed by applying these videos as model inputs. In [15], the classification of strokes was performed using two-dimensional joint coordinates and a graph showing the relationship between scores and strokes, focusing on the Olympic games. In this study, the classified strokes were used to analyze actual games. In addition, in [16], a dataset called P^2^A was created. P^2^A is a dataset created from videos of the World Championships and the Olympics, and labels were attached to classify and detect 14 types of behavior, including strokes. In addition, the authors of [17] conducted research on table tennis video analysis with the creation of datasets. In this work, a network called TTNet, designed for event detection and player segmentation, was proposed, and OpenTTGames was created and published. OpenTTGames is a dataset created from videos taken by the authors, and the positions of the balls and players are labeled. Since these datasets [5,16,17] contain actual videos of matches, the analysis considers videos taken in various situations and is not limited to a specific experimental environment.

As demonstrated above, there are many studies on the classification of strokes in table tennis. However, many of them assume specific experimental environments; for example, they involve attaching a sensor to a player or installing a camera on a table tennis table. It is difficult to apply these methods to actual game videos. There are also studies on the classification of game videos, but these are rare. In this study, we propose a stroke classification method for game videos.

## 3. Proposed Method

This section describes the proposed stroke classification method and its preparation. In this study, we classify table tennis strokes through a multi-label classification task by assigning two labels to each stroke, demonstrating the strong similarity between the classes.

In the following subsections, we describe the videos for classification, the multi-labeling method for stroke classes, and the network structure for the multi-label classification task.

### 3.1. Dataset

**Figure 3:**
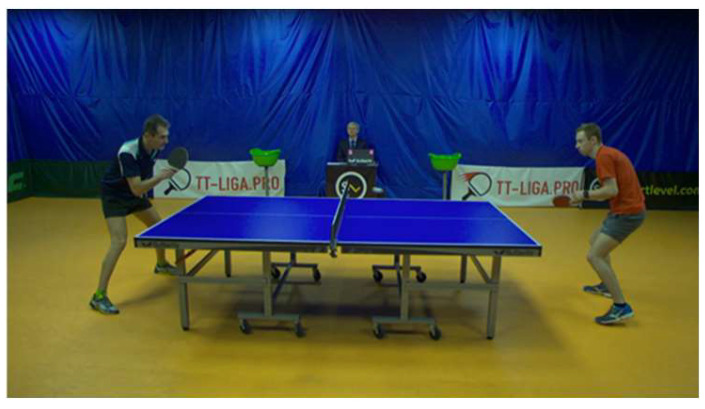
Videos for classification: OpenTTGames is a video dataset of games captured from outside the competition area.

As mentioned above, most previous studies have prepared a specific experimental environment. In this study, we classify strokes using actual game videos. As the game video data, we use OpenTTGames, which was created and published in [17]. OpenTTGames is a dataset consisting of 12 different games and includes the strokes of 24 players in total. One frame used in this study is shown in Figure 3; this is a video shot of the entire game from the side of the table tennis table. Since occlusions of the body and arms may occur, this type of classification is more difficult compared to videos in which the player is captured from the front. In this study, we classify strokes using a dataset of game videos taken from a viewpoint in which the filming does not disturb the game.

Next, the labeling process is described. OpenTTGames is a dataset created for tasks such as ball detection. Since stroke labels are not assigned, one of the present authors, who is an experienced table tennis player, performed the labeling. The types of classes and the amount of data for each class are shown in Table 1. Various scales can be considered for the number of classes to be classified. In this study, labels were assigned to 10 classes, as defined in the literature [1]. First, the strokes are divided into two types—forehand and backhand—according to the position at which the returning ball flies. Then, the strokes are classified into five types—topspin, push, block, flick, and lob—according to the direction of rotation and the speed in the hitting ball. However, since OpenTTGames does not specifically focus on stroke collection, the amount of data for each class is biased, and the data are divided into eight classes, excluding “forehand lob” and “backhand lob”. Examples of the videos for each of the eight classes are shown in Figure A1 in Appendix A.

**Figure 4:**
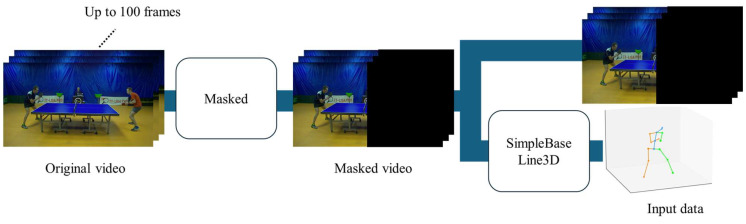
Preparing the data: masking the side of the player who does not hit the ball in black.

Next, the procedure for the creation of a dataset from the video is described. The steps followed to extract joint coordinates from the original video are shown in Figure 4. First, a video of up to 100 frames is prepared, in which the time during which a stroke is performed is defined as the time at which the ball touches the opponent’s platform to the time at which the ball is hit back and touches the opponent’s platform. OpenTTGames consists of videos taken at 120 fps, and the duration of the strokes considered in this study is very short, i.e., less than 1 s. Furthermore, since we use videos of games, areas not related to the stroke are present in the frame. Therefore, the image is divided into the left and right sides, with the table tennis table between them, and the side at which the stroke is not performed is regarded as irrelevant to the classification task and is masked in black. Next, as input for the action recognition model, the joint coordinates of the player are obtained so as to use not only the video, but also the joint coordinates as inputs. Since there may be occlusions of the body and arms, the indirect coordinates are three-dimensional. To obtain the coordinates, a three-dimensional posture estimation method named SimpleBaseLine3D [18] is used, and the coordinates of 17 joints defined in Human 3.6M [19] are obtained. One piece of data consists of a video for 100 frames and the joint coordinates. If the stroke duration defined above is less than 100 frames, the value of the remaining time is set to 0.

### 3.2. Network Structure for Classification

**Figure 5:**
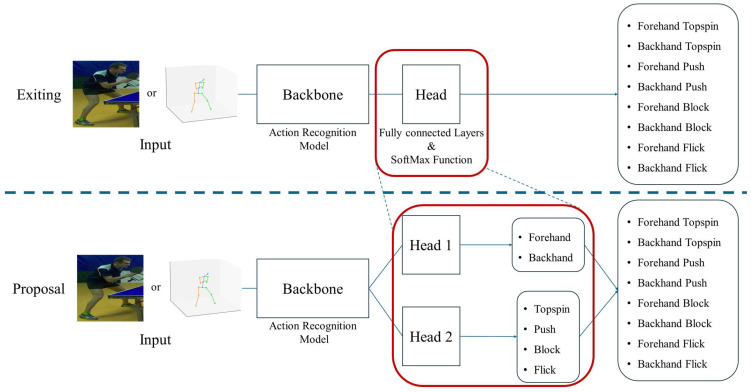
Network structure: (**top**) stroke classification using existing behavior recognition models; (**bottom**) stroke classification using multi-labeling. Head is divided into Head 1 and Head 2, and two classification results are output.

Next, the network structure for the classification of strokes via a multi-label classification task is described. The schematic diagram is shown in Figure 5. The upper part is a network structure for the classification of eight classes at a time, and the lower part is a network structure for the classification of two classes and four classes.

First, the network for the classification of eight classes at a time is described, as shown at the top of Figure 5. Generally, the structure of an action recognition model is divided into two parts: the backbone and the head. The backbone determines the features present in the input information. The used structures depend on the model, but three-dimensional convolutional layers are often used when videos are input, and two-dimensional convolutional layers are often used when joint coordinates are input, considering the joint coordinates as a graph structure. The head deforms the features obtained in the backbone to match the shape of the output. Specifically, the head includes fully connected layers that match the features with multiple dimensions to the structure of the output, as well as a SoftMax function to calculate the probability of the class being classified. In this way, the action recognition model represents a network that determines the action that is taken for a given input. In this work, video or three-dimensional joint coordinates are used as input data. First, the feature values of the video or three-dimensional joint coordinates are calculated using the action recognition model as the backbone. Here, we do not propose a network structure for feature computation, but use various existing action recognition models as the backbone. The specific models used will be described in Section 4. Then, the calculated features are input into the following head part to obtain the classification result. The head part includes the fully connected layer and the SoftMax function; it shapes the features identified in the backbone and calculates the class probability. Action recognition using video or joint coordinates is not limited to table tennis, but most models have a similar process, in which feature values are determined for input and classification probabilities are obtained from these feature values. Therefore, a similar process can be used for stroke classification.

Next, a network for the performance of two classification tasks—two-class and four-class classification—is described. However, the general process is the same as in the network structure for eight-class classification. The two classification tasks are implemented by bifurcating the head associated with the backbone into two parts. In the conventional action recognition model, which obtains one output for one input, a single backbone and a head are included. In the proposed method, the attributes of two strokes are classified by two heads from the features obtained by the same backbone. Head 1 classifies the two classes of forehand/backhand. Forehand/backhand is determined by whether the ball is hit with the racket facing the ball and the palm facing forward or with the racket facing the ball and the back of the hand facing forward. Therefore, it is determined independently of the rotation of the ball. Head 2 concerns the four classes of topspin/push/block/flick. These four classes are determined by the direction of rotation and the speed of the ball, and they can be identified independently of the forehand/backhand class. Therefore, in this network, they can be determined independently of the result of Head 1. The final stroke class is determined from the combination of the classes obtained in Head 1 and Head 2. For example, when the class of “forehand” is obtained in Head 1 and the class of “topspin” is obtained in Head 2, the final classification is obtained as “forehand topspin”. As described above, the number of classes to be classified is reduced by dividing into the classification tasks across the two heads, and the classification accuracy is expected to be improved in comparison to classification using the conventional single-output action recognition model. In addition, since the network structure is changed only through the branching of the head part, the proposed method can be easily applied to well-known action recognition models.

The weight is updated via the loss of the two outputs or the average of them. The loss used here is the cross-entropy loss, which is widely used in classification tasks. The equation for the cross-entropy loss is shown in Equation (1), where *x* is the class, *p*(*x*) is the probability distribution of the correct answer, and *q*(*x*) is the estimated probability distribution.

(1) $$H\left(p,\;q\right)=-\sum_{x}p\left(x\right)\log\left(q\left(x\right)\right)$$

Since Head 1 and Head 2, shown in the lower part of Figure 5, are applied only in two-class classification and four-class classification, the weights are updated using the cross-entropy loss obtained from each output. Equations (2) and (3) show the respective calculation formulas. Equation (2) contains *H*_2*classes*_, which is the loss calculated from the two-class classification of forehand/backhand, and it considers the probability *p* of the correct answer and the estimated probability *q* for each of the two classes. Equation (3) contains *H*_4*classes*_, which is the loss calculated from the four-class classification of topspin/push/block/flick, and it considers the probability *p* of the correct answer and the estimated probability *q* for each of the four classes.

(2) $$H_{2classses}\left(p,\;q\right)=-\left(p_{forehand}\log\left(q_{forehand}\right)+p_{backhand}\log\left(q_{backhand}\right)\right)$$

(3) $$H_{4classses}\left(p,\;q\right)=-\left(p_{topspin}\log\left(q_{topspin}\right)+p_{push}\log\left(q_{push}\right)+p_{block}\log\left(q_{block}\right)+p_{flick}\log\left(q_{flick}\right)\right)$$

On the other hand, the backbone is associated with two classification tasks, so both outputs must be reflected. However, in this method, in which we perform the final classification by combining the two classification tasks, the loss for the final eight-class classification cannot be obtained. Therefore, the weight is updated using the average value of the losses of the two outputs, as shown in Equation (4). $H_{2classes}$ and $H_{4classes}$, shown in Equations (2) and (3), are the losses obtained from the results of two-class classification and four-class classification, respectively, and the average is taken to calculate the loss $H_{all}$ to update the backbone.

(4) $$H_{all}\left(p,\;q\right)=\frac{1}{2}\left(H_{2classes}\left(p,\;q\right)+H_{4classes}\left(p,\;q\right)\right)$$

### 3.3. Multi-Labeling

Next, we describe the multi-labeling of stroke classes. In this method, strokes that originally had one label are divided and given two labels to create a multi-label classification task. In table tennis, a stroke is a technique used to hit the ball, and it is classified from various perspectives, such as the direction of the body in approaching the ball, the speed and direction of rotation, and the velocity of the ball. Forehand/backhand is the most significant type of classification here. Forehand/backhand is determined by whether the ball is hit with the palm facing the incoming ball or the back of the hand facing the incoming ball. Therefore, the stroke is determined independently of how the ball is spun. Another type of classification is based on the hit ball. The stroke is classified in various ways according to the direction of rotation and the speed of the ball. It should be noted here that the same hit ball exists for both forehand and backhand. For example, as shown in rows 2 and 3 of Table 1, the rotation called “topspin” is present in both forehand and backhand.

**Figure 6:**
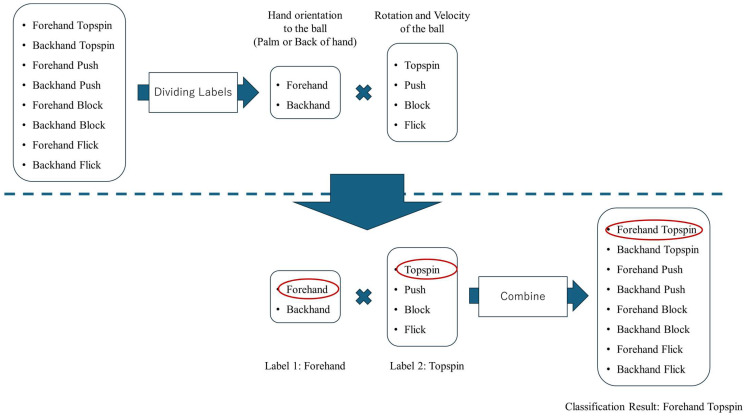
Process of splitting labels and retrieving final classification results from split labels.

Thus, the strokes in table tennis can be roughly divided into two types: One is determined by the player’s posture, and the other is determined by the ball being hit. These two factors can be determined independently of each other. Therefore, two types of labels are assigned to a stroke, and the stroke is classified according to their combination. Figure 6 shows the process of label assignment and the process of classifying the final stroke based on the classification results regarding the divided labels. The part above the dotted line in Figure 6 shows the process of assigning labels.

Labels are assigned according to the player’s posture, consisting of two classes, i.e., forehand and backhand, as described above. The characteristics of each class are described below.

- Forehand: hitting the ball with the face of the racket, where the palm faces the incoming ball, and returning the ball;
- Backhand: hitting the ball with the face of the racket, where the back of the hand faces the incoming ball, and returning the ball.

On the other hand, in the classification based on the hit ball, various hitting styles are defined according to the classification level. In this study, following a previous study [1], the strokes are classified into four types: topspin, push, block, and flick. The characteristics of each type are as follows.

- Topspin: applying upward rotation to the ball by swinging the racket up from the bottom;
- Push: returning a ball that falls on the table by pushing it forward;
- Block: adjusting the angle of the racket to minimize movement and returning a ball that falls on the table;
- Flick: applying upward rotation to a ball that falls on the table by quickly moving the wrist.

The stroke is determined by combining these two classification results. Instead of performing eight-class classification in one instance, we divide it into two tasks, with two classes and four classes; thus, while the number of tasks increases, the difficulty of each task decreases. For example, as shown in the lower part of Figure 6, if the classification result based on the direction of the hand towards the ball is “forehand” and the classification result regarding the rotation or velocity of the hit ball is “topspin”, the stroke is classified as “forehand topspin”.

## 4. Experiment

This section describes the experiment in which we compare the accuracy of the proposed method, which involves classifying strokes in a multi-label classification problem, with the existing action recognition models; we use videos or joint coordinates as inputs. The dataset used for classification is the one described in Section 3.1. This dataset contains a total of 792 strokes in 8 classes, as shown in Table 1. To evaluate the classification accuracy for the strokes of unknown players, we divide the dataset into two parts. The amount of data after division is shown in Table 2. The original data refer to 24 players, and we divide them into two parts for testing: data A, which contain 626 strokes by 16 players, and data B, which contain 166 strokes by 8 players. In other words, separate data are allocated for training/validation and testing. Data A are divided at a ratio of 7:3 for the learning/verification process. By dividing the data in this way, we evaluate the generalizability of the method in classifying strokes that are associated with individual differences and habits among different players.

The learning was performed using an NVIDIA GeForce RTX3080 with 10 GB memory. The detailed experimental environment is described in Table 3.

The learning was performed using parameters tuned by Optuna [20] for each action recognition model used. Table 4 shows the tuned hyperparameters and their search ranges. The four hyperparameters that were tuned were the batch size, optimization function, weight decay, and learning rate. The learning rate was fixed during learning, and the learning process was performed for 400 epochs. The hyperparameters after tuning are shown in Table A1 in Appendix B.

### 4.1. Evaluation Metrics

Next, the evaluation indices are described. Two major evaluation indices, the accuracy and F1 score, are used. To calculate the accuracy and F1 score, the recall and precision must be determined. The following describes the accuracy and F1 score, and the values required to calculate them.

#### 4.1.1. Recall and Precision

When classifying a class *A*, the recall indicates the number of items whose correct class is *A* can be classified as class *A*, and the precision indicates the number of items classified as class A that have the correct class *A*. The calculation is performed using Equations (5) and (6) below.

(5) $$Recall=\frac{TP}{TP+FN}$$

(6) $$Precision=\frac{TP}{TP+FP}$$

- The terms *TP*, *FP*, *TN*, and *FN* have the following meanings.
- *TP*: the number of items classified as class *A* whose correct class was class *A*;
- *FP*: the number of items classified as class *A* whose correct class was not class *A*;
- *TN*: the number of items not classified as class *A* whose correct class was not class *A*;
- *FN*: the number of items not classified as class *A* whose correct class was class *A.*

#### 4.1.2. Accuracy and F1 Score

The accuracy is the percentage of all classification results that can be classified correctly and is calculated using Equation (7).

(7) $$Accuracy=\frac{TP+TN}{TP+FP+TN+FN}$$

The *F1* score is calculated using Equation (8). It is the harmonic mean of the recall and precision. From these values, the accuracy is calculated. It indicates the percentage of all classification results that are correctly classified. There are several types of *F1* scores, such as the *F1*, which is typically used for binary classification, and the *macro-F1* and *weighted-F1*, which are typically used for multi-class classification. The *F1* score is calculated using Equation (8). It is obtained via the harmonic mean of the recall and precision in each class. The macro-f1 is calculated using Equation (9) and is the average of the *F1* for each class. In Equation (9), n indicates the number of classes. In this case, n = 8.

(8) $$F1=2\times \frac{Recall\times Precicion}{Recall+Precision}$$

(9) $$Macro-F1=\frac{1}{n}\sum_{k=1}^{n}{F1}_{k}$$

The accuracy is the percentage of correct answers, while the *macro-F1* is the average value of the *F1* for each class. Thus, it is an index that is not affected by changes in accuracy for classes with fewer data. The *weighted-F1*, a variant of the *macro-F1*, is the average of the *F1* scores weighted for each class and is expressed as in Equation (10).

(10) $$Weighted-F1=\frac{1}{n}\sum_{k=1}^{n}w_{k}\times {F1}_{k}$$

In Equation (10), *w_k_* denotes the weight in class *k*. In this work, we assign the weight to the inverse of the amount of data in each class. We adopt this metric as it is more sensitive to the classification of small data in datasets with large biases.

### 4.2. Backbone Models

Next, we describe the existing action recognition models used as the backbones. Firstly, we use TSTCNN [11], which was proposed specifically for stroke classification in table tennis. We also use MMAction2 [21] as an example of a general action recognition model. MMAction2 is an open-source toolbox for video understanding tasks; it provides various models and methods for action recognition and detection, using video/joint coordinates as input. We also use five models—C3D [22], I3D [23], C2D [24], R(2+1)D [25], STGCN [26], and AGCN [27]—to conduct action recognition tasks using video/joint coordinates as input. The features of each model are described below.

- TSTCNN [11]

TSTCNN is a network in which an attention mechanism is added to a 3D convolutional layer to handle video and optical flows. In the classification of the data in TTStroke-21, which is a dataset consisting of table tennis strokes, it was shown to be more accurate than other well-known action recognition models [23] when using three-dimensional convolution.

- C3D [22]

C3D was the first model to use three-dimensional convolutional layers. While image learning has previously been performed with a two-dimensional convolutional layer, this model seeks to learn the feature values of videos by performing convolution along the time axis, and it has become the basis of several subsequent action recognition models using three-dimensional convolution.

- I3D [23]

I3D is a model that uses three-dimensional convolutional layers based on the expansion of two-dimensional convolutional layers. Since it is designed by inflating two-dimensional convolutional layers, it can learn more efficiently by utilizing the weights of existing two-dimensional convolutional neural networks.

- C2D [24]

C2D is a model that considers a video as a collection of images and extracts the feature via the two-dimensional convolution of each image. After extracting the features from each frame individually, the weighted average is calculated to capture the temporal features between frames. Since the convolution is two-dimensional, it is relatively lightweight.

- R(2+1)D [25]

R(2+1)D is a model that divides conventional three-dimensional convolution into a two-dimensional convolution process that handles spatial information and a one-dimensional convolution process that handles temporal information. Since these layers convolve each type of information individually, it is possible to extract the features independently.

- STGCN [26]

STGCN is a model that considers the human skeleton as a graph structure, with joint coordinates as nodes and the connections between the joints as edges. It extracts the features via graph convolution. By processing the spatial/temporal connections as a graph, the expressiveness is greatly improved compared to the conventional networks that focus on time series information. The graph structure of the skeleton proposed in this study has been adopted in many skeleton-based action recognition models.

- AGCN [27]

AGCN is a model that adaptively changes the graph structure of the joints in the model. Learning an end-to-end parameterized joint graph in addition to the joint graph prepared as input offers greater flexibility compared to the conventional GCN, demonstrating strong versatility across various types of data.

### 4.3. Experimental Results

Next, the experimental results are described. Firstly, Table 5 and Table 6 show the values of each evaluation index according to the presence or absence of multi-labeling in the verification/test data for each backbone, using video as input. In these tables, the precision and recall are the average values of each class. As shown in Table 5, multi-labeling improved the classification accuracy for the validation data in all five backbones. In addition, the F1 score showed an improvement in four backbones: TSTCNN, C3D, I3D, and R(2+1)D. On the other hand, as shown in Table 6, the accuracy was improved in three backbones: TSTCNN, C3D, and R(2+1)D. There were cases in which the F1 value was both worsened and improved depending on the backbone. TSTCNN, C3D, C2D, and R(2+1)D had improved macro-F1 values, and C3D, C2D, and R(2+1)D had improved weighted-F1 values. Among the cases in which the macro-F1 was improved, the values of both precision and recall were improved, with the exception of the test data of R(2+1)D. This indicates that the classification accuracy for each class was improved, and the classification was not biased towards a particular class with a large amount of data. In addition, the accuracy of TSTCNN was 63.9%, while that of the other action recognition models was significantly lower, at 22.9~39.8%. Therefore, it is very difficult to classify strokes that do not exist in the learning data when using a general action recognition model. This is because there are individual differences in strokes among players, and it is difficult to achieve generalization as the results depend on these differences. In summary, in the action recognition model that uses video as input, the stroke classification method with multi-labeling showed an improvement in 13 out of 15 evaluation items (precision and recall are excluded because they are values used to calculate the F1) on the validation data, as well as improvements in 10 items on the test data. Thus, in the stroke classification model that uses video as input, the classification accuracy was improved to a certain extent by multi-labeling. On the other hand, since the accuracy and F1 scores of the test data were significantly lower compared to those of the validation data, it can be concluded that the classification of the strokes of unknown players is difficult when videos are used. This is because there are individual differences among players, even in the same types of strokes. The stroke is determined by the speed and rotation of the ball, but the stroke style varies depending on the player’s tactics (e.g., offensive or defensive), habits, and physique. Therefore, even in the same type of stroke, it may not be possible to obtain consistent results; thus, the classification results for the strokes of unknown players are extremely low.

Table 7 and Table 8 show the values of each evaluation index for the backbones with and without multi-labeling in the validation/test data, when using joint coordinates as input. As shown in Table 7, multi-labeling improved the classification accuracy for the validation data in both backbones. In addition, the F1 score was improved in three out of four items, and only the weighted-F1 in STGCN decreased. On the other hand, as shown in Table 8, the classification accuracy was improved in both backbones, and the F1 score was also improved across all items for the test data. Based on the above results, stroke classification with multi-labeling was performed in the action recognition model, using joint coordinates as input. For the validation data, an improvement was observed in five out of six evaluation items, and, for the test data, an improvement was observed in all six items. Thus, in stroke classification using joint coordinates, multi-labeling enables improved accuracy.

In addition, when the evaluation was performed using videos and joint coordinates as input, the accuracy of the video inputs was the highest, at 70.6%/63.9% for the validation/test data, respectively, while that for joint coordinates was 87.7%/69.3%. Moreover, the macro-F1 reached a maximum of 0.679/0.410 when videos were used as input and 0.770/0.507 when joint coordinates were used as input. According to the above, joint coordinates are more effective for classification regarding both indices. The reason for this is that table tennis involves swinging the arms; thus, joint coordinates, which directly reflect these movements as values, can better capture such features. Additionally, in this study, we focus on game videos, which may cause occlusion related to players’ bodies. For example, part of the arm may be hidden when using the video itself, but 3D joint coordinates can be used to indicate that the hidden arm is located at the back of the body. This further explains why the use of three-dimensional joint coordinates is effective for the classification of game videos.

**Figure 7:**
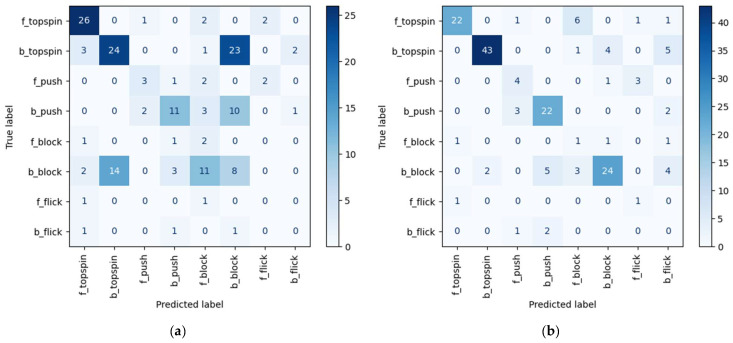
Confusion matrix of classification results when using STGCN as backbone: (**a**) Without multi-labeling, (**b**) With multi-labeling.

Next, the details of the classification are described. In this section, we discuss the results obtained with the test data when using STGCN, where the improvement in the accuracy was particularly remarkable. In STGCN, when performing classification with multi-labeling, the accuracy was improved by 2.1% and 18.1% for the validation and test data, respectively. For the test data, the accuracy was improved the most among the action recognition models tested. Figure 7 shows the confusion matrix of the classification results for the test data without and with multi-labeling in STGCN. Figure 7a,b show the results obtained without multi-labeling and with multi-labeling, respectively. The vertical axis shows the true label, the horizontal axis shows the predicted label, and the number in the box where each label name matches is the number of correct classifications. In the classification without multi-labeling, it can be seen in Figure 7a that many strokes are classified as a backhand block when the correct class is backhand topspin or as backhand topspin when the correct class is backhand block. This is likely because the backhand topspin and backhand block exhibit similar features. As noted in Section 3.2, topspin is a method of hitting by swinging the racket up from the bottom to spin, but backhand topspin involves swinging the arm to a lesser extent than in forehand topspin. Therefore, it is similar to the block movement, which involves little swinging of the arm, leading to misclassification. On the other hand, as shown in Figure 7b, the number of errors was greatly reduced in the multi-label classification task. It is considered that, by classifying topspin and block independently of forehand/backhand, it was possible to observe large and small variations in arm swing among the topspin movements. For the same reason, the number of instances in which backhand push was misclassified as backhand block also decreased. Moreover, the number of strokes in which backhand block was misclassified as forehand block decreased significantly. The reason for this is that, in contrast to the above finding, it was possible to observe the differences in posture by classifying forehand/backhand independently, even in blocks with small arm swings.

**Figure 8:**
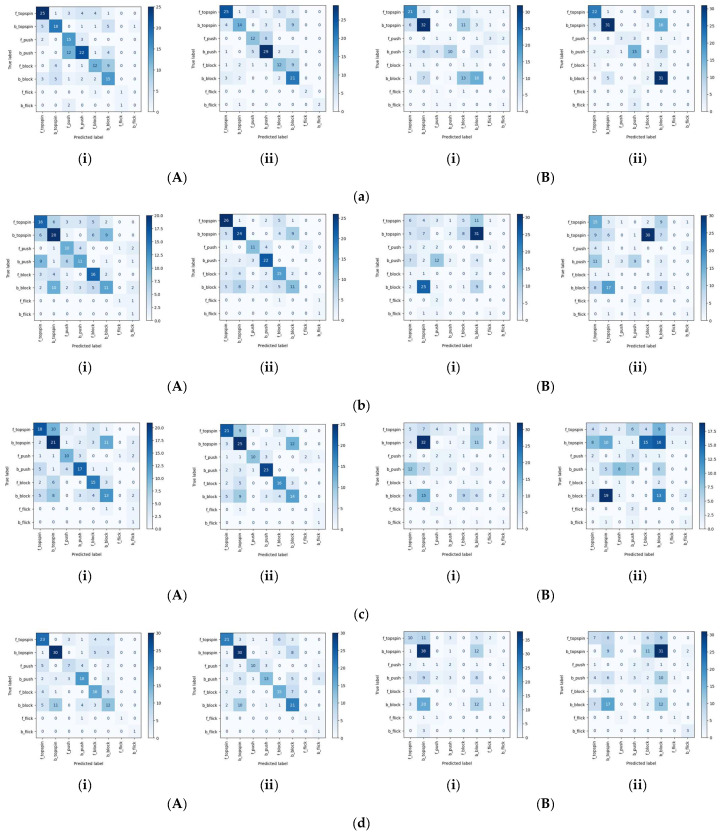

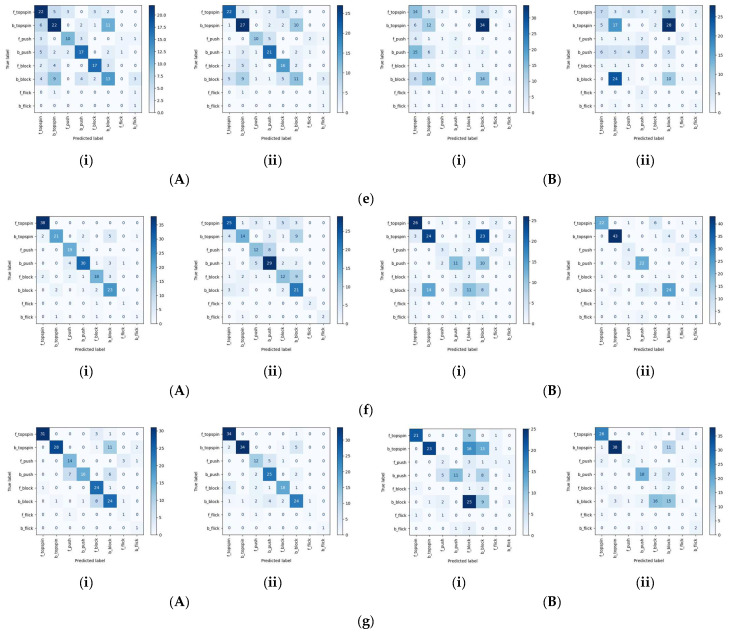
Confusion matrix of classification results for each action recognition model: (**a**) TSTCNN, (**b**) C3D, (**c**) I3D, (**d**) C2D, (**e**) R(2+1)D, (**f**) STGCN, (**g**) AGCN, (A) Validation, (B) Test, (i) Without multi-labeling, (ii) With multi-labeling.

The confusion matrices of the classification results obtained with other behavioral recognition models are listed in Figure 7 and Figure 8. In both cases, as with STGCN, backhand topspin and backhand block are often misclassified. This tendency is especially evident in classification using videos as input, as shown in Figure 8a–e. Meanwhile, in classification using three-dimensional joint coordinates as input, as shown in Figure 8f,g, backhand topspin and backhand block are correctly classified as compared to the use of videos as input. These results show that it is especially difficult to classify similar movements during the classification of table tennis strokes, and three-dimensional joint coordinates are more suitable inputs in such cases.

## 5. Conclusions

In this study, we propose a multi-label classification method for stroke classification in table tennis. In table tennis, as the basic action of swinging the arm is similar, stroke classification is more difficult than in general action recognition tasks. Therefore, we sought to reduce the difficulty of each task and improve the classification accuracy by assigning two types of labels according to the attitude of the player who hits the ball and the hit ball, followed by dividing the task into multiple action recognition tasks. Multi-label classification can be easily implemented with various models by branching the head part of the output, using the existing action recognition model as the backbone. In the experiment, it was confirmed that the accuracy was improved for many action recognition models. In addition, when we used two types of input—video and three-dimensional joint coordinates—it was confirmed that the latter yielded superior accuracy and effectiveness.

However, there are still several problems in the classification of table tennis strokes. The first is data bias. Since the videos used in this study were not designed for stroke classification, there was a bias in the number of strokes performed. There were some strokes that involved frequent hitting and others that did not; thus, it is necessary to collect and experiment with a wider variety of strokes for improved learning. Therefore, we are considering the use, creation, and expansion of datasets containing larger numbers of strokes. The second problem is the accuracy regarding unknown data. In this study, we inserted the strokes of players who did not exist into the learning data as test data and evaluated the classification accuracy. However, the accuracy decreased significantly for the validation data—it was reduced by up to 18.4%. Since the classification of the strokes of unknown players is highly important in the analysis of videos of actual games, it is necessary to improve the accuracy in this context. We aim to achieve this by using features that are more consistent among players and by using and proposing models with high levels of generalization. The third problem is end-to-end and real-time classification. At present, the acquisition of joint coordinates from videos is conducted separately from the classification of the strokes, and the obtained data are used as input data. Therefore, it is not possible to classify the strokes in an end-to-end manner from videos at present. To improve the practicality, we aim to develop a consistent system that obtains joint coordinates and classifies movements in an end-to-end manner. In the future, by solving these three issues, we hope to achieve improved accuracy and practicability.

## Figures and Tables

**Table 1:** The names of the classes and the amount of data for each.

	Class Name	Amount of Data
1	Forehand Topspin	155
2	Backhand Topspin	171
3	Forehand Push	65
4	Backhand Push	141
5	Forehand Block	87
6	Backhand Block	148
7	Forehand Flick	10
8	Backhand Flick	15
9	Forehand Lob	0
10	Backhand Lob	0
	Total	792

**Table 2:** Amount of data after division.

	Use	Amount of Data	Number of Players
Data A	train/validation	626	16
Data B	test	166	8

**Table 3:** Experimental environment (hardware).

Hardware Environment	
CPU	Intel (R) Core (TM) i7-13700KF
GPU	NVIDIA GeForce RTX 3080 (NVIDIA, Santa Clara, CA, USA)
RAM	64 GB
GPU Memory	10 GB

**Table 4:** Parameters tuned by Optuna and search ranges.

Hyperparameter	Search Range
Batch Size	1~8
Optimizer	SGD, Adam, AdamW
Weight Decay	1 × 10^−3^~1 × 10^−10^
Learning Rate	1 × 10^−1^~1 × 10^−5^

**Table 5:** Results of action recognition models using videos as input (validation data).

Backbone	Multi-Labeling	Accuracy	Precision	Recall	Macro-F1	Weighted-F1
TSTCNN [11]	no	69.0	0.499	0.506	0.496	0.354
	yes	70.6	0.715	0.666	0.679	0.855
C3D [22]	no	54.8	0.433	0.537	0.440	0.350
	yes	62.2	0.506	0.572	0.524	0.463
I3D [23]	no	55.3	0.421	0.513	0.424	0.196
	yes	59.0	0.520	0.570	0.519	0.369
C2D [24]	no	64.9	0.670	0.605	0.625	0.846
	yes	66.0	0.631	0.624	0.568	0.416
R(2+1)D [25]	no	55.9	0.460	0.540	0.461	0.238
	yes	59.0	0.476	0.560	0.478	0.240

**Table 6:** Results of action recognition models using videos as input (test data).

Backbone	Multi-Labeling	Accuracy	Precision	Recall	Macro-F1	Weighted-F1
TSTCNN [11]	no	53.0	0.386	0.348	0.347	0.145
	yes	63.9	0.444	0.411	0.410	0.131
C3D [22]	no	16.9	0.134	0.111	0.102	0.028
	yes	22.9	0.210	0.184	0.186	0.100
I3D [23]	no	36.7	0.195	0.233	0.195	0.107
	yes	18.7	0.168	0.172	0.162	0.086
C2D [24]	no	39.8	0.221	0.199	0.197	0.044
	yes	32.5	0.289	0.322	0.265	0.389
R(2+1)D [25]	no	28.3	0.200	0.187	0.174	0.077
	yes	28.9	0.199	0.191	0.185	0.091

**Table 7:** Results of action recognition models using skeletons as input (validation data).

Backbone	Multi-Labeling	Accuracy	Precision	Recall	Macro-F1	Weighted-F1
STGCN [26]	no	85.6	0.732	0.718	0.718	0.529
	yes	87.7	0.732	0.745	0.729	0.443
AGCN [27]	no	83.5	0.648	0.749	0.648	0.409
	yes	85.1	0.774	0.773	0.770	0.824

**Table 8:** Results of action recognition models using skeletons as input (test data).

Backbone	Multi-Labeling	Accuracy	Precision	Recall	Macro-F1	Weighted-F1
STGCN [26]	no	51.2	0.353	0.348	0.326	0.115
	yes	69.3	0.521	0.527	0.507	0.246
AGCN [27]	no	57.8	0.410	0.282	0.306	0.075
	yes	62.7	0.482	0.466	0.459	0.222

## Data Availability

Restrictions apply to the datasets because the data are part of an ongoing study.

## Appendix A

The specific frames of the video used in this study, described in Section 3, are shown in Figure A1. Figure A1 contains one stroke example for each of the eight classes. In each image, the player on the left hits the ball.

## Appendix B

Each backbone was optimized via Optuna, and the hyperparameters used are shown in Table A1.

**Table A1:** Optimized hyperparameters.

Backbone	Batch Size	Optimizer	Weight Decay	Learning Rate
TSTCNN [11]	7	AdamW	5.60 × 10^−9^	2.07 × 10^−4^
C3D [22]	7	Adam	2.39 × 10^−8^	4.69 × 10^−4^
I3D [23]	8	Adam	1.68 × 10^−6^	4.31 × 10^−3^
C2D [24]	6	SGD	3.92 × 10^−9^	1.56 × 10^−5^
R(2+1)D [25]	7	SGD	1.05 × 10^−9^	4.57 × 10^−5^
STGCN [26]	3	Adam	4.96 × 10^−10^	1.14 × 10^−2^
AGCN [27]	2	Adam	5.21 × 10^−10^	6.93 × 10^−4^
